# Reverse-phase phosphoproteome analysis (RPMA) of signaling pathways induced by HTLV-1 infection

**DOI:** 10.1186/1742-4690-8-S1-A177

**Published:** 2011-06-06

**Authors:** Taissia G Popova, Aarthi Narayanan, Lance Liotta, Emanuel F Petricoin III, Charles Bailey, Kylene Kehn-Hall, Fatah Kashanchi

**Affiliations:** 1 George Mason University, Department of Molecular and Microbiology, National Center for Biodefense and Infectious Diseases, Manassas, VA, 20110, USA; 2 George Mason University, Center for Applied Proteomics and Molecular Medicine, Manassas, Virginia, 20110, USA; 3 The George Washington University Medical Center, Department of Microbiology, Immunology, and Tropical Medicine, Washington, DC, 20037, USA

## 

Phosphorylation plays a key role in regulating many signaling pathways. Cell fate decisions in response to extracellular agents, including pathogenic invaders, are commonly mediated by phosphorylation-regulated signaling cascades that transduce signals into stimulus-specific actions, such as changes in gene expression pattern. Here we utilized a novel approach where we infected cells with HTLV wild type and mutant clones and analyzed the phosphorylation status of the cells. For phosphoproteomic analysis the cell lysates were printed onto nitrocellulose membrane slides. Each slide was then probed with one of 360 different antibodies specific against phosphorylated or total forms of signaling proteins. The antibodies were selected to monitor the molecular networks involved in host responses most likely affected by virus exposure, namely survival, apoptosis, inflammation, growth, differentiation, and immune response. This RPMA technique was extensively validated in our previous studies with regard to specificity of antibodies, sensitivity, and accuracy of phosphoprotein detection in cell lysates. Changes in NFkB, SAP/JNK, ERK and AKT and against phosphorylated form of p-38 and PTEN will be discussed.

